# Epitheliocystis Distribution and Characterization in Brown Trout (*Salmo trutta*) from the Headwaters of Two Major European Rivers, the Rhine and Rhone

**DOI:** 10.3389/fphys.2016.00131

**Published:** 2016-04-18

**Authors:** Maricruz Guevara Soto, Lloyd Vaughan, Helmut Segner, Thomas Wahli, Beatriz Vidondo, Heike Schmidt-Posthaus

**Affiliations:** ^1^Department of Infectious Diseases and Pathobiology, Centre of Fish and Wildlife Health, University of BernBern, Switzerland; ^2^Department of Pathobiology, Institute of Veterinary Pathology, University of ZurichZurich, Switzerland; ^3^Department of Clinical Research and Veterinary Public Health, Veterinary Public Health Institute, University of BernBern, Switzerland

**Keywords:** *Candidatus* Piscichlamydia salmonis, *Candidatus* Clavichlamydia salmonicola, *Candidatus* Similichlamydia sp., epitheliocystis, survey, Switzerland, catchment

## Abstract

We present a first description of the distribution and characterization of epitheliocystis infections in brown trout (*Salmo trutta*) from the upper catchments of two major European rivers, the Rhine and the Rhone. Overall, epitheliocystis was widely distributed, with 70% of the Rhine and 67% of the Rhone sites harboring epitheliocystis positive brown trout. The epitheliocystis agents *Candidatus* Piscichlamydia salmonis and *Candidatus* Clavichlamydia salmonicola could be identified in both catchments, although their relative proportions differed from site to site. Additionally, in two rivers in the Rhine catchment, a new species of *Candidatus* Similichlamydia was identified. Based on the histology, infection intensity, and severity of pathological changes were significantly more pronounced in mixed chlamydial infections, whereas single infections showed only low numbers of cysts and mild pathology. Infections could be found over a wide range of temperatures, which showed no correlation to infection prevalence or intensity.

## Introduction

Epitheliocystis (EP) is a disease name describing an intracellular bacterial infection of finfish gill and skin epithelia, resulting in hypertrophy of host cells (Hoffman et al., 1969; Paperna and Sabnai, 1980; Desser et al., 1988; Lewis et al., 1992; Nowak and LaPatra, 2006). Nowadays, EP has been described worldwide in over 90 different species of wild and cultured marine and fresh water fish (Corsaro and Greub, 2006; Nowak and LaPatra, 2006; Stride et al., 2013a,b). The causative agents mostly belong to the phylum *Chlamydiae*, but also include γ- and β-proteobacteria (Kurahashi and Yokota, 2007; Toenshoff et al., 2012; Mendoza et al., 2013; Katharios et al., 2015; Seth-Smith et al., 2016). In salmonids, *Candidatus* Piscichlamydia salmonis (Draghi et al., 2004) and *Candidatus* Clavichlamydia salmonicola (Karlsen et al., 2008) were identified so far and these bacterial species seem to be specific for salmonids and were not identified in any other fish so far. According to our knowledge, all epitheliocystis agents are quite host-specific. In addition to farmed marine salmon (*Salmo salar)* (Draghi et al., 2004), *Ca*. P. salmonis has also been found in Arctic char (*Salvelinus alpinus*) farmed in fresh water (Draghi et al., 2010). In contrast, *Ca.* C. salmonicola appears to be fresh water specific. It was found in farmed salmon and in wild brown trout (*Salmo trutta*) (Karlsen et al., 2008; Mitchell et al., 2010; Schmidt-Posthaus et al., 2012), and disappeared upon transfer of infected salmon to marine cages (Mitchell et al., 2010).

In the Northern hemisphere, cultured salmonids appear to be prone to EP with infections reported for juvenile steelhead trout (*Oncorhynchus mykiss*) (Rourke et al., 1984), cultured lake trout (*Salvelinus namaycush*) (Bradley et al., 1988), Atlantic salmon (*S. salar* L.) (Nylund et al., 1998; Draghi et al., 2004; Karlsen et al., 2008; Mitchell et al., 2010; Steinum et al., 2010) and Arctic char (*Salvelinus alpinus*) (Draghi et al., 2007, 2010). While infections are mostly well tolerated in older fish (Schmidt-Posthaus et al., 2001, 2012), high mortalities have been described in larval and juvenile stages (Miyaki et al., 1998; Draghi et al., 2004; Nowak and LaPatra, 2006; Katharios et al., 2008; Mitchell and Rodger, 2011). The reservoir for these *Chlamydiae* infections is still unclear. Even though amoebae have been postulated as a reservoir (Corsaro and Greub, 2006), EP agents could not be cultivated neither in amoebae nor in any other system. An alternative environmental reservoir could be widely spread wild fish populations, like wild brown trout (*S. trutta*).

The presence of EP has been described sporadically in wild brown trout in Norwegian rivers (Karlsen et al., 2008) and in river catchments of the (sub) alpine regions of Switzerland (Schmidt-Posthaus et al., 2001, 2012). Although a native salmonid species abundant throughout Europe and into Asia, it can be divided into *Salmo trutta trutta*, an anadromous or ocean migrating population and into nonanadromous *Salmo trutta fario* or potamodromous *Salmo trutta lacustris* forms, which are local resident river populations, which may also migrate into adjoining lakes. Although attractive as putative reservoir populations, there is nevertheless no systematic overview on the distribution, prevalence and abundance of EP causing bacteria in wild brown trout populations. In this context, it would be especially interesting to compare brown trout populations of the Rhine catchments, which theoretically at least, could exchange with salmonid populations of the North Sea with geographically separate brown trout populations of the upper Rhone, which ultimately flows into the salmonid poor Mediterranean. For these reasons, our aim in this present study was to provide the first overview on the occurrence of EP in resident wild brown trout populations (*Salmo trutta fario*) of rivers in the upper reaches of the Rhine and Rhone. The study examines the geographic distribution of EP in Swiss brown trout, along with prevalence, infection intensity, occurrence at different temperatures, and possible pathological lesions associated with the infection. Additionally, the bacterial genotypes in the two catchments were compared to investigate the interspecies relationship between agents found in the Rhine and the Rhone catchments and their relationship to agents found in the North Sea.

## Materials and methods

### Study catchments

**Figure 1:**
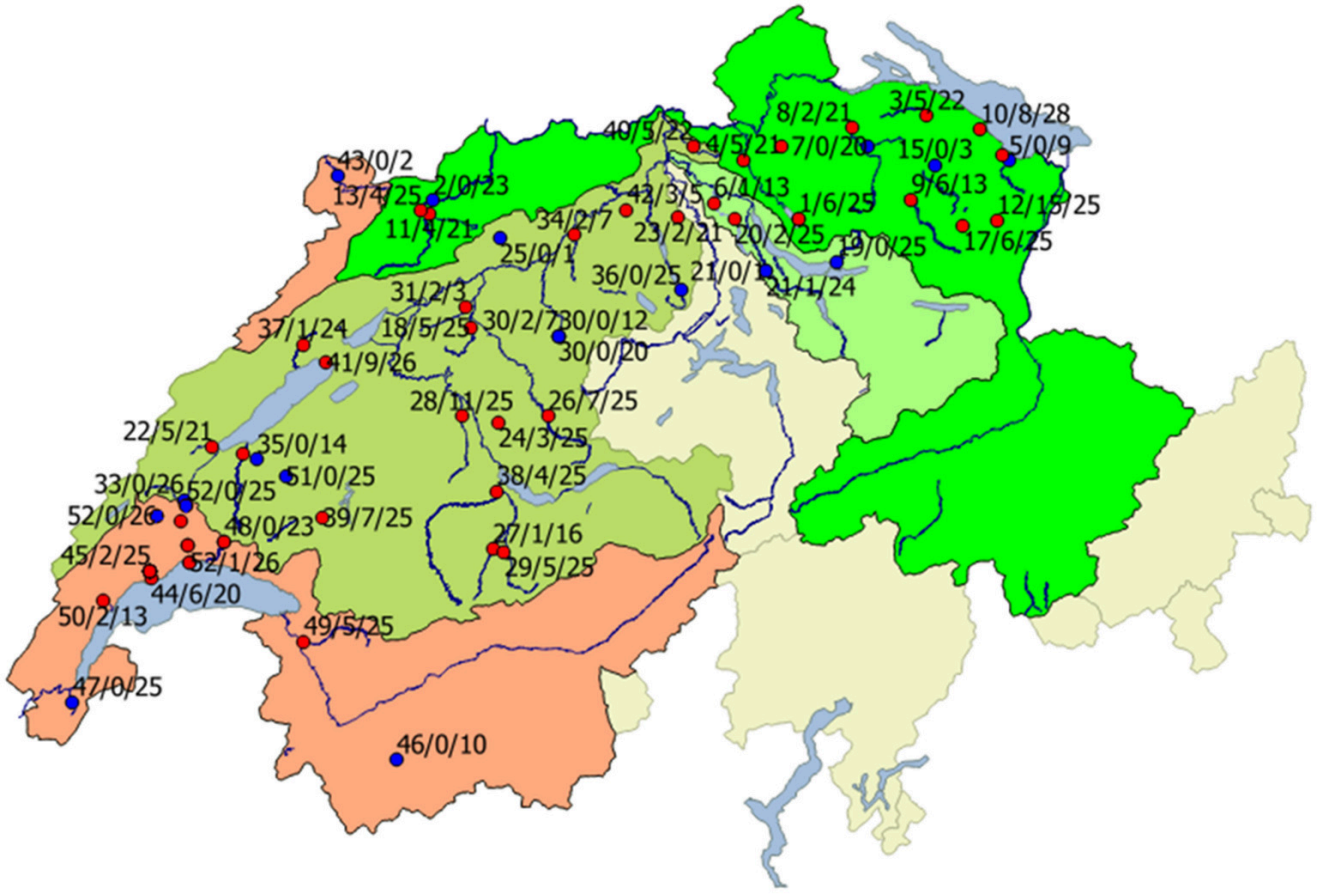
**Geographical distribution of infected (red) and non-infected sites (blue) in Rhine (green areas) and Rhone (red area) catchments**. Red, Rhone region; bright green, Rhine region; light green, Limmat region; dark green, Aare region. Beside the locations, the river number (names and according numbers are given in Table 1), number of infected animals, and total number of investigated animals is given.

From June to November 2012, wild young-of-the-year (YOY) brown trout were collected in 52 rivers, whereby 42 rivers belonged to the Rhine catchment and 10 rivers to the Rhone catchment (Figure 1). The sampling took place in the frame of a nationwide project aiming to assess the health status of trout in Swiss rivers. In most of the rivers, one sampling site was selected, but in 6 rivers up to 7 sampling sites along the course of the rivers were included. In total, 46 sampling sites were included from the Rhine catchment, 18 sampling sites belonged to the Rhone. Brown trout were sampled by electrofishing over a stretch of 100 m at each site.

### Sample collection

At each sampling site we aimed to investigate 25 YOY brown trout, however in a number of river sites this number could not be achieved because of low population numbers. A total of 1442 brown trout were examined from 64 different sampling sites. The fish collected were euthanized in the field by an overdose of tricaine methanesulfonate (MS-222®, Argent Chemical Laboratories, Redmont, USA). Each fish was investigated for external lesions, and the length was measured. Fish were placed in left lateral position for necropsy and the left operculum was removed. The first left gill arch was fixed in 10% buffered formalin for histological examination and the second arch was preserved in RNAlater (Sigma-Aldrich, Missouri, USA) for polymerase chain reaction (PCR) and sequencing.

### Water temperature

At most sampling sites, water temperature was measured once at each sampling site at the date of sampling.

### Histopathology

Formalin fixed gill samples were trimmed, embedded in paraffin, and sections of 4 μm thickness were cut. Sections were stained with hematoxylin and eosin (H&E). Gill sections were examined by light microscopy for the presence of cysts, and the cyst number per gill arch was counted (infection intensity). As all fish were belonging to the same age class (YOY), the size of the gill arch was comparable between different animals. Pathological lesions like edema, inflammation, and lamellar fusion were graded as 0 (no lesions), 1 (mild pathology), 2 (moderate pathology) to 3 (severe pathology).

The cyst morphology was used to characterize different types of bacteria (see also Guevara Soto et al., 2016). *Ca*. P. salmonis like cysts (type 1) were characterized by a dark basophilic amorphous center and clear surrounding halo, up to 10 μm in diameter. *Ca*. C. salmonicola like cysts (type 2) were histologically visible as lightly basophilic granulated cysts, slightly bigger, up to 15 μm in diameter. Mixed infections (type 3) showed cysts of both types on one gill arch. This classification based on morphology was used for all statistical investigations.

### 16SrRNA gene PCR and sequencing

For PCR and sequencing, a subset of locations was selected form the Rhine and the Rhone catchment. In the Rhine catchment, the Aabach, Buenz, Emme, Glatt, and Kander were investigated, from the Rhone, the Aubonne, Boiron, and Venoge. For the Rhine catchment, locations were randomly selected out of a pool of locations where we were able to obtain well-preserved samples in RNAlater. For the Rhone subset, data were obtained from Guevara Soto et al. (2016).

DNA from gills from RNAlater was extracted using the QIAGEN DNAeasy Blood and Tissue kit (Qiagen, Hilden, Germany) and stored at −20°C until use.

PCR was performed initially to generate a 290 bp Chlamydial signature sequence using the following primers 16SigF: 5′-CGG CGT GGA TGA GGC AT-3′ and 16Sig R: 5′-TCA GTC CCA GTG TTG GC-3′ (Everett et al., 1999). Positive gill samples from the first screening were partly further investigated by 16S rRNA gene PCR to generate longer fragments of 1089 or 1520 bp using primers 16SigF 5′- TCA GTC CCA GTG TTG GC-3′ in combination with VR-1 rev 5′-GATAAGGGTTGCGCTCGTTG-3′ (1089 bp) or 16SB1 rev 5′- TAC GGY TAC CTT GTT ACG ACT T -3′ (1520 bp).

All PCR reactions were performed in individual 50 μL reaction mixtures containing 26.7 μl water, 5 μl PCR Buffer Roche® 10 × (MgCl_2_ 20 mM), 5 μl MgCl_2_ (25 mM), 5 μl dNTP (10 mM), 2.5 μl each primer (10 μM), 5 μl DNA, and 0.8 μl Fast Star Taq (Roche, Basel, Switzerland).

The signature amplification used 45 cycles with annealing at 54°C and extension at 72°C for 90 s. The 1089 and 1520 bp amplifications were the same but with an annealing temperature of 52°C. PCR products were visualized after agarose electrophoresis by a BioDoc-IT Imaging System UVP.

Samples that showed positive results for the 1089 or 1520 bp products were purified using the MinElute PCR Purification Kit (Qiagen, Hilden, Germany) and immediately cloned using the TOPO TA Cloning® Kit (pCR®2.1-TOPO® vector) (Invitrogen, California, USA) and One Shot TOP10 chemically competent *E. coli* (Invitrogen, California, USA).

Plasmids from individual clones were purified using the QIAPrep Spin Miniprep Kit (Qiagen, Hilden, Germany) and those with the amplicon inserts were identified using Eco RI (Biolabs, Massachusetts, USA) according to manufacturer's instructions. Positive clones were capillary sequenced by Microsynth (Balgach, Switzerland). The resulting reads were assembled and alignments prepared using CLC Main Workbench 7.6.4. (CLC bio, Qiagen) and compared with published data using blastn against the Genbank database. Novel 1089 bp sequences of *Ca*. Similichlamydia sp. are available in ENA-EMBL under the accession numbers LT222046–LT222048.

### Statistical analysis

For the statistical analyses, a subset of sample sites was selected, with only one sampling site per river to avoid bias because of varying repeated measures per river. Additionally, sampling sites with only very low numbers of brown trout (*n* < 9) were also excluded from the statistical analyses. In total, 45 rivers were selected for the statistics, 36 from the Rhine catchment and 9 from the Rhone catchment. We calculated the point prevalence from each river as the percentage of infected fish per total number of investigated animals at the time point of sampling. To evaluate differences in prevalence in both catchments, four river systems (see below) and temperature were calculated by means of logistic regression models. The river system categorical variables included Aare, Limmat, Rhine, and Rhone, this last one as the reference category.

The number of cysts per gill arch was not normally distributed. So, differences in the number of cysts per gill arch (infection intensity) between catchments (Rhone and Rhine), the type of pathological lesions (edema, inflammation, lamellar fusion), the type of cyst morphology, and temperature were explored by means of non-parametric Kruskal-Wallis rank sum tests. All analyses were carried out in R, version 3.1 (https://www.r-project.org/) and packages rcmdr, car, Rcmdmisc.

## Results

### Distribution of EP infections

The geographical distribution of sites harboring infected and non-infected brown trout in both catchments is shown in Figure 1. In the Rhine catchment, 33 of 42 rivers were positive for brown trout with EP infections, while in the Rhone catchment 6 of 10 rivers, sometimes with several sampling sites, showed brown trout with bacterial cysts on the gills (Figure 1). From a total of 875 fish collected in the Rhine 161 were positive, while in the Rhone 567 brown trout were investigated with 47 EP positive animals. The Rhine catchment was further subdivided in three main tributary regions, the Aare, Limmat, and the Rhine. In the Aare region, 18 of 24 river sites harbored epitheliocystis positive brown trout, in the Limmat 2 out of 4 investigated sites and in the Rhine there were 13 out of 18 investigated river sites positive. In the Rhone 12 out of 18 river sites showed EP positive animals.

### Prevalence and infection intensity per catchment

**Figure 2:**
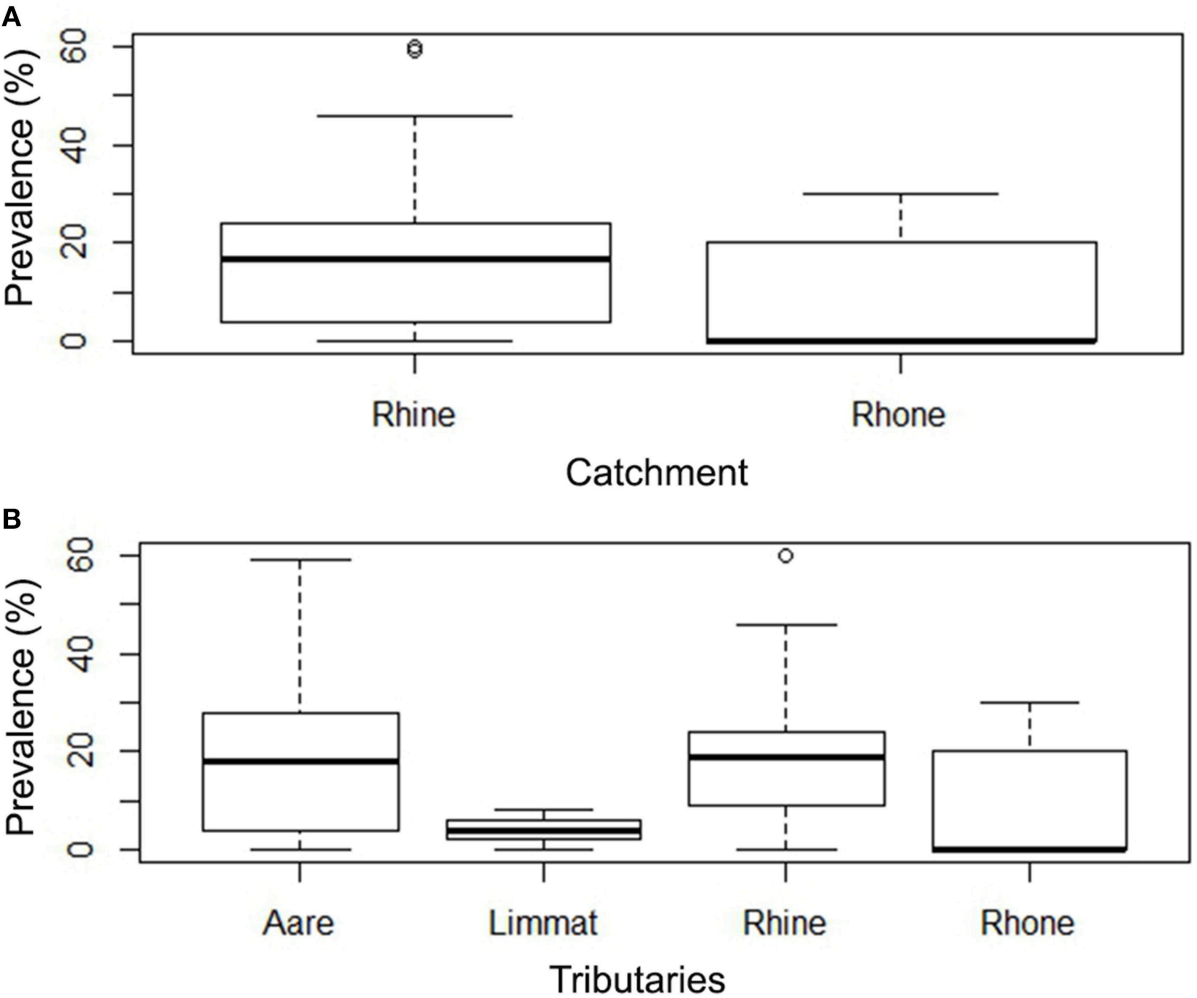
**(A)** Box Plot of the prevalence (%) of EP in Rhine and Rhone catchments. There is a higher probability of finding infected animals in the Rhine catchment compared to the Rhone catchment (Two-level Logistic Regression model, *p* < 0.000000). **(B)** Prevalence of infected animals in tributaries (Aare, Limmat, and Rhine belonging to the Rhine catchment; Rhone belonging to the Rhone catchment).

To evaluate prevalence and intensity data, a subset of sampling sites (*n* = 45) (total of 987 investigated animals) was used (inclusion criteria are described in Sections Materials and Methods, Statistical Analysis). Based on these data, 29 rivers (80%) of the Rhine catchment revealed to be positive for EP, while in the Rhone catchment 5 rivers (55.6%) showed brown trout with bacterial cysts in the gills. There is a higher probability of finding infected animals in the Rhine catchment compared to the Rhone catchment (Logistic Regression model, *P* = 0.00506). The comparison of the different river systems rendered only two effects, with evidence of higher infection risk in the Rhine (*P* = 0.00195), followed by the Aare (*P* = 0.00400). The Limmat showed no increased risk compared to the Rhone (*P* = 0.13602). Within the positive sites, the prevalence of infected animals per sampling site varied from 4 to 60%, with most sites showing prevalences of < 30% (Figures 2A,B, Table 1).

**Table 1:** **Overview of epiheliocystis in brown trout in the two catchments Rhine and Rhone, shown are catchment area, investigated river, region these rivers belong to, number shown in Figure 1, number of investigated animals (n), sampling date, water temperature measured on the sampling date, epitheliocystis prevalence (%), and number of positive brown trout per sample, infection intensity calculated by number of cysts per gill arch and bacteria morphology classified in three classes**.

**Catchment**	**River**	**Region**	**Number**	**N**	**Sampling date**	**T (°C)**	**Prevalence (%) (n positive animals)**	**Intensity (n cysts) median, 5th, 95th percentile**	**Bacteria morphology**
Rhine	Aabach	Rhine	1	9	04.09.12	17.7	0 (0)	0	0^*^
	Birs	Rhine	2	23	22.08.12	15.5	0 (0)	0	0
	Brine	Aare	22	21	July 2012	16.8	24 (5)	1,1,3.6	1,2
	Buenz	Aare	23	21	26.09.12	14.1	10 (2)	8.5,4.45,13	2,3^*^
	Chemibach	Rhine	3	22	11.09.12	14.9	23 (5)	7,1.8,60	2,3
	Chise	Aare	24	25	9.10.12	13.7	12 (3)	5,3.2,17	1,3
	Emme	Aare	26	25	20.08.12	16	28 (7)	3,1,11	1,2,3^*^
	Engstlige	Aare	27	16	26.11.12	5.5	6 (1)	26	3
	Glatt	Rhine	4	21	20.08.12	21.2	20 (5)	2,1,65	1,2,3
	Guerbe	Aare	28	25	09.10.12	17.4	44 (11)	8,2.5,138	1,2,3
	HohliAa	Limmat	6	13	04.10.12	18.6	8 (1)	1	1
	Jona	Limmat	19	25	19.10.12	15.5	0 (0)	0	0
	Kander	Aare	29	25	25.10.12	8.5	20 (5)	4,1,29	2,3^*^
	Langeten	Aare	30	12	22.08.12	19.9	0 (0)	0	0
	Lauche	Rhine	7	20	24.10.12	10.8	0 (0)	0	0
	Menthue	Aare	32	22	20.09.12	11	59 (13)	3,1,11	2,3
	Murg	Rhine	8	21	24.10.12	13.5	10 (2)	4,2.2,6	2
	Necker	Rhine	9	13	27.08.12	20	46 (6)	4.5,2.25,9	1,2,3
	Nozon	Aare	33	26	22.08.12	NI	0 (0)	0	0
	Reppisch	Limmat	20	25	18.09.12	18.0	8 (2)	1.5,1.05,2	1
	Ron	Aare	36	25	05.09.12	16.3	0 (0)	0	0
	Ruisseau de Vaux	Aare	35	14	June 2012	NI	0 (0)	0	0
	Salmsacher Aach	Rhine	10	28	11.09.12	13	29 (8)	14,2,320	2,3
	Scheulte	Rhine	11	21	31.08.12	17.1	19 (4)	8,5.3,264	2,3
	Seyon	Aare	37	24	28.08.12	16.3	4 (1)	25	3
	Sihl	Limmat	21	24	03.10.12	17.7	4 (1)	55	3
	Simme	Aare	38	25	11.09.12	10.2	16 (4)	3,1.15,4	2
	Sionge	Aare	39	25	05.09.12	16	28 (7)	15,4.4,85	1,2,3
	Sitter	Rhine	12	25	20.10.12	8.7	60 (15)	5,1,23	2,3
	Sorne	Rhine	13	25	31.08.12	15	16 (4)	14,2.15,47	2,3
	Steinach	Rhine	14	24	28.08.12	20	17 (4)	7,1.75,26	1,2,3
	Surb	Aare	40	22	18.09.12	16.5	23 (5)	2,1,36	1,2,3
	Thielle	Aare	41	26	20.09.12	NI	35 (9)	5,2,100	1,2,3
	Toess	Rhine	16	26	10.09.12	18.6	19 (5)	2,1,28	1,2,3
	Urnaesch	Rhine	17	25	28.08.12	21	24 (6)	24,3.25,128	2,3
	Urtenen	Aare	18	25	11.09.12	16.4	20 (5)	3,1.4,6	1,2,3
Rhone	Aubonne	Rhone	44	20	11.09.12	15.8	30 (6)	6,1.25,125.3	1,2,3^*^
	Boiron	Rhone	45	25	10.07.12	18.6	0 (0)	0	0^*^
	Drance	Rhone	46	10	30.10.12	NI	0 (0)	0	0
	Drize	Rhone	47	25	June 2012	NI	0 (0)	0	0
	Flon de Carrouge	Rhone	48	25	June 2012	NI	24 (6)	4.5,2.25,13	1,2,3
	Grande-Eau	Rhone	49	25	17.10.12	NI	20 (5)	1,1,3	2,3
	Promenthouse	Rhone	50	13	11.09.12	15.3	15 (2)	6,1.5,11	2,3
	Ruisseau de Seigneux	Rhone	51	25	June 2012	NI	0 (0)	0	0
	Venoge	Rhone	52	25	17.07.12	17.9	0 (0)	0	0^*^

Shown is the subset of samples used for the statistical analyses. Bacteria morphology: three types were histologically distinguished: 1: indicative for Ca. P. salmonis, 2: indicative for Ca. C. salmonicola, 3: Mixed infections with both cyst types;

* indicates locations which were randomly selected for sequencing; n = number of animals.

Regarding the geographical distribution, there was no clustering visible; sites with high prevalence and infection intensities were located nearby sites with few positive brown trout or negative sites (see Figure 1).

**Figure 3:**
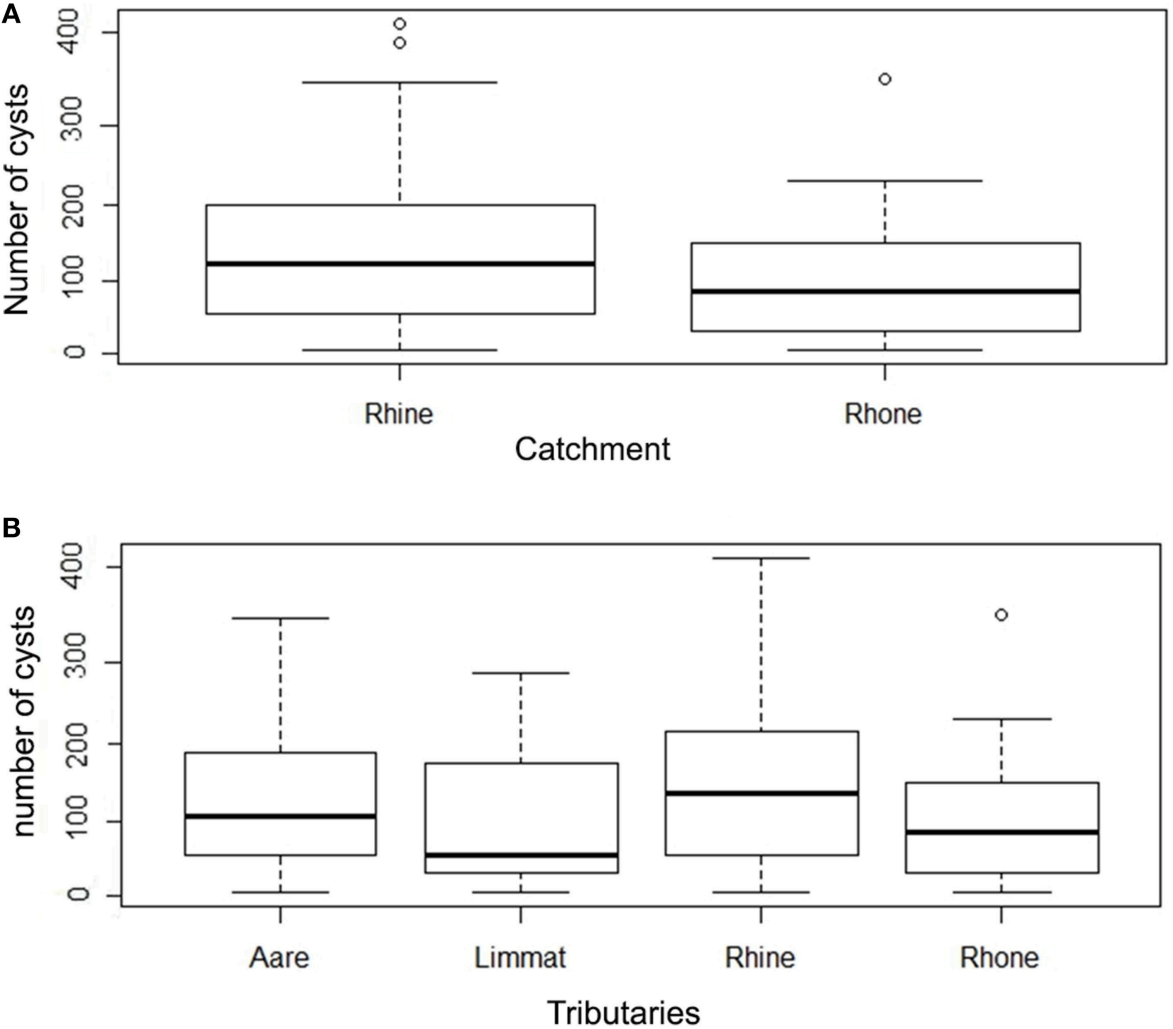
**(A)** Box plot of number of cysts per gill arch in Rhine and Rhone catchment. **(B)** Number of cysts per gill arch in tributaries (Aare, Limmat, and Rhine belonging to the Rhine catchment; Rhone belonging to the Rhone catchment) (note the logarithmic scale in the y axis).

The infection intensity, as a measure of the number of cysts per gill arch, is also highly skewed toward small values (Figure 3, Table 1). Most of the infected fish (143 out of 165) had fewer than 50 cysts per gill arch. In the Rhine catchment, there are some outliers with up to 450 cysts per gill arch, mainly in the Rhine region (Figure 3). However, infection intensity showed no significant differences between the catchments or the tributaries (*P* = 0.2336; Kruskal-Wallis rank sum test).

### Morphologies of epitheliocystis lesions

**Figure 4:**
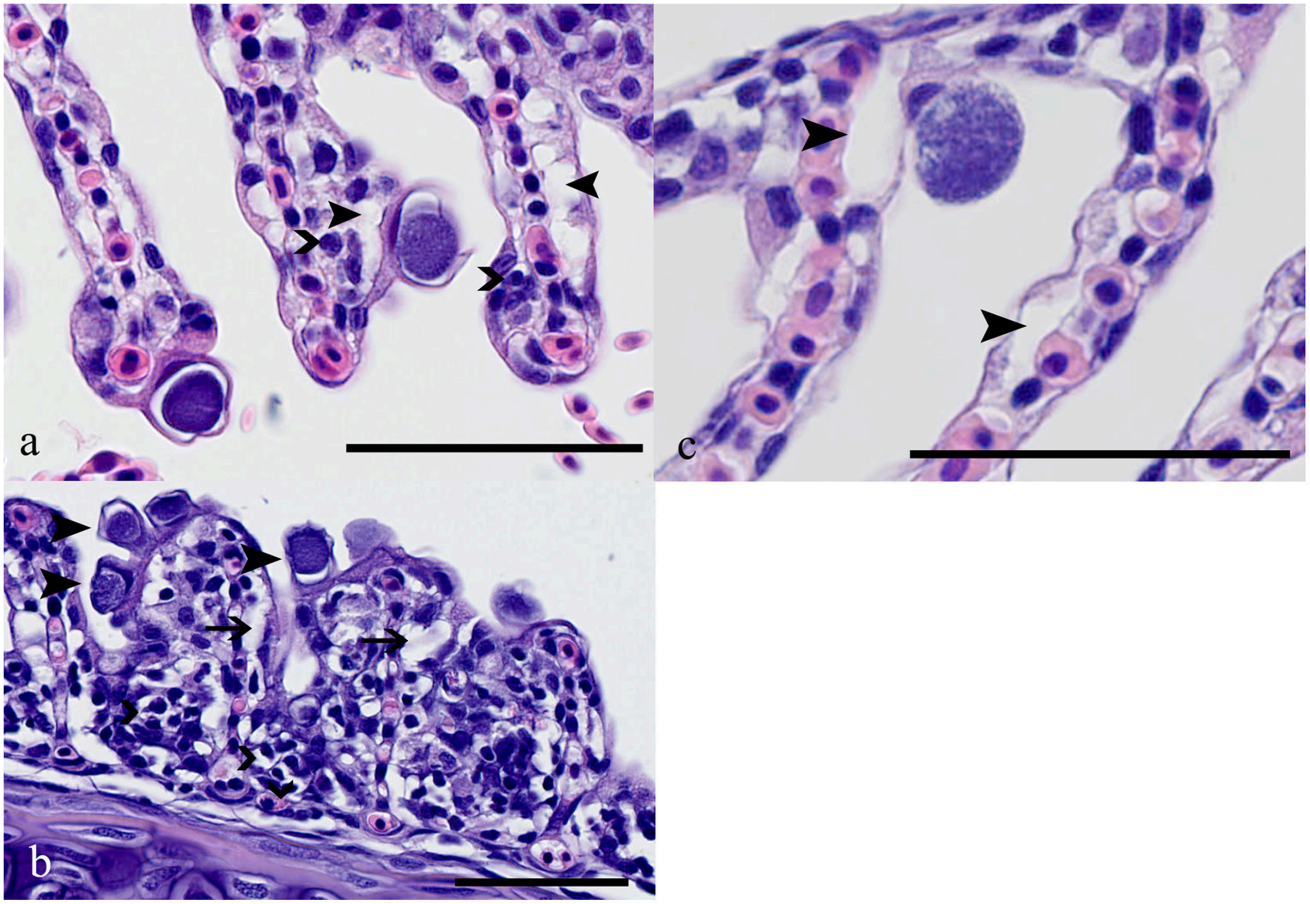
**(A)** Brown trout (Salmo trutta), gills, Ca. P. salmonis cysts characterized by condensed basophilic intracellular material surrounded by a clear halo; up to 20 μm in diameter. Closed arrowheads indicate edema in the subepithelial area, open arrowheads indicate scattered infiltration with mainly lymphocytes. HE, bar = 50 μm. **(B)** Ca. P. salmonis cysts (e.g., arrowheads), lamellae showing fusion, infiltration with lymphocytes, macrophages, and eosinophilic granular cells (open arrowheads) and subepithelial edema (arrows). HE, bar = 50 μm. **(C)** Ca. C. salmonicola cyst characterized by granular loosely arranged material, up to 20 μm in diameter. In the surrounding tissue only scattered edema (closed arrowheads) is visible. HE, bar = 50 μm.

Two types of inclusion morphologies were identified, both leading to hypertrophy of host epithelial cells. Histologically, the first inclusion was characterized by compact dark basophilic central bacteria with formation of a clear halo around the bacterial cyst, leading to margination of the host cell nucleus (Figure 4A). This morphology is attributable to *Ca*. P. salmonis (type 1) (Schmidt-Posthaus et al., 2012; Guevara Soto et al., 2016). The second cyst type was histologically characterized by granular, loosely arranged basophilic bacterial material, with the host cell nucleus mostly not visible (Figure 4C), representing *Ca*. C. salmonicola (type 2) (Schmidt-Posthaus et al., 2012; Guevara Soto et al., 2016). Cysts of both morphologies were also present on the same gill arch identified as mixed infection (type 3). The different morphologies permit a reliable distinction between *Ca*. P. salmonis and *Ca*. C. salmonicola, and form the basis for the following analyses.

**Figure 5:**
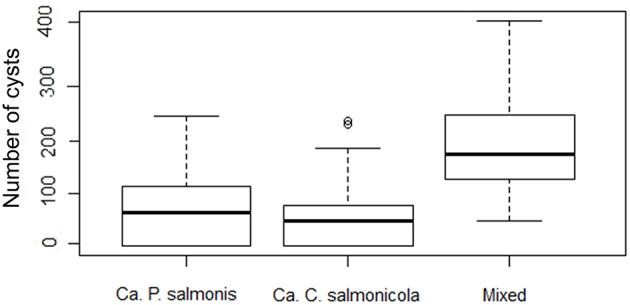
**Box Plot of morphology type and number of cysts per gill arch (infection intensity) (note the logarithmic scale in the y axis)**.

Mixed infections were encountered most frequently, while single infections with *Ca*. C. salmonicola were found least often (Figure 5). The significant association between the mixed infections and the number of cysts per gill arch indicates that infection intensity is higher in mixed infection compared to single infection with either of the two bacterial types (Kruskal-Wallis rank sum test, *P* < 0.0000).

### Pathology

**Figure 6:**
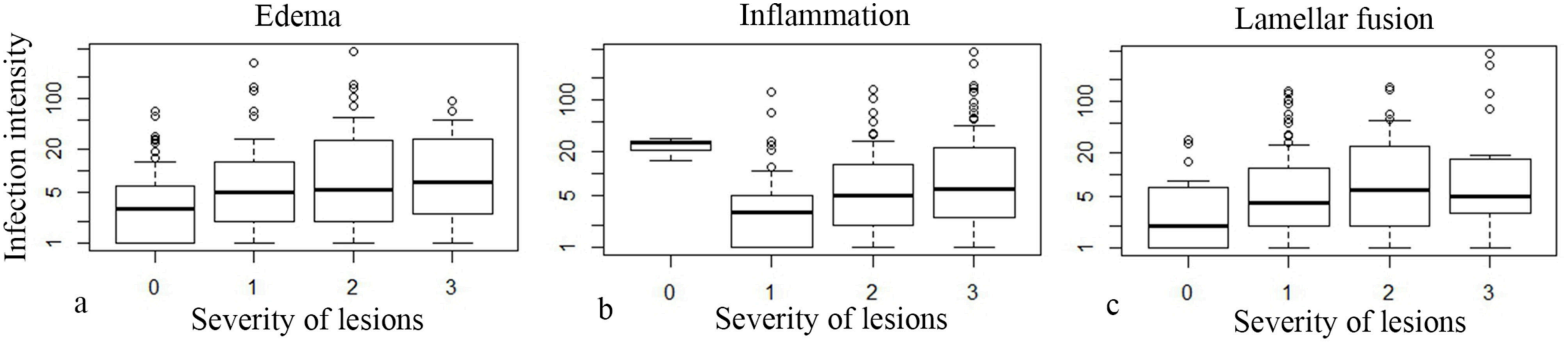
**Box Plots of severity of pathological lesions (edema, infiltration, lamellar fusion) and number of cysts per gill arch (infection intensity) (note the logarithmic scale in the y axis)**.

*Ca*. P. salmonis infection usually showed a mild to moderate epithelial cell hyperplasia, mild edema and infiltration with mainly lymphocytes (Figure 4B), while *Ca*. C. salmonicola infection was only rarely associated with a host reaction (Figure 4C). Infection intensity (number of cysts per gill arch) was weakly associated with some of the pathological changes (Kruskal-Wallis rank sum tests: *P* = 0.052, *P* = 0.0011, *P* = 0.1609, edema, inflammation, and lamellar fusion, respectively) (Figure 6).

### Associations with site temperature

**Figure 7:**
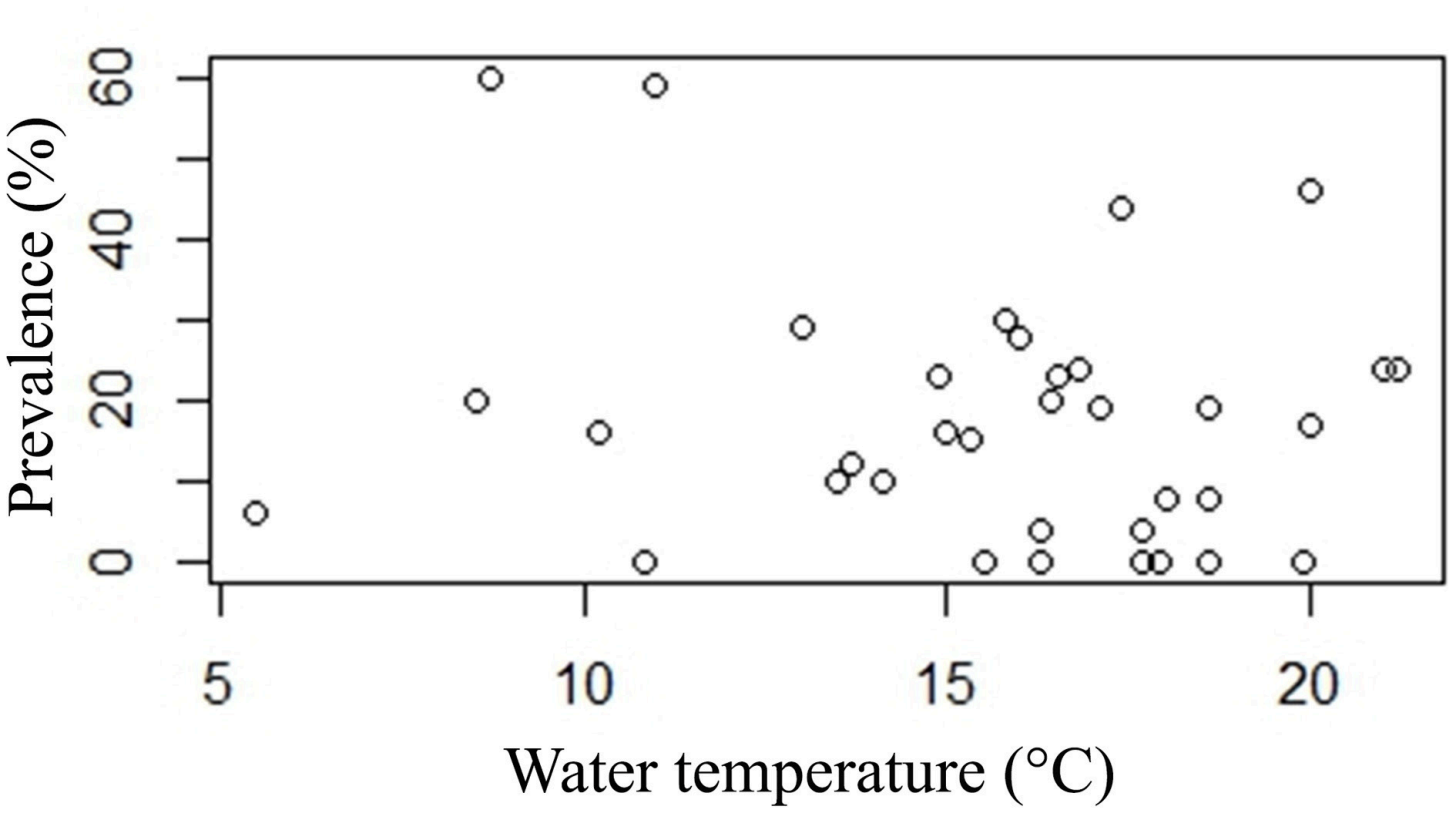
**Plot of prevalence point data (%) and water temperature (°C)**.

Neither prevalence nor infection intensity was associated with site temperature. EP was present at a wide range of temperatures from 5.5 to 21.2°C. Even high prevalence (>30% prevalence) is present at disparate temperatures (8.7–20°C) (Figure 7).

### Sequencing data

For PCR and sequencing, a subset of locations was selected from the Rhine and the Rhone catchments. In the Rhine catchment, the Aabach, Buenz, Emme, Glatt, and Kander were investigated, from the Rhone, the Aubonne, Boiron, and Venoge were selected. For the Rhone subset, data were obtained from Guevara Soto et al. (2016). Nearly full length (1089 bp) or full length (1520 bp) products could not always be obtained, whereas the 290 bp chlamydial signature sequence amplification was a robust method for screening, although this short sequence has limited value for detailed phylogenetic analysis. Comparing this common 290 bp chlamydial signature sequence region of all amplificates, 89% of all sequences obtained were found to be 99–100% identical to published sequences (Draghi et al., 2004; Karlsen et al., 2008; Guevara Soto et al., 2016) of either *Ca*. P. salmonis (66/133) or *Ca*. C. salmonicola (52/133). The remaining 11% (only found in two sites, Emme and Buenz) were divided between three closely related sequences, all 97% identical to published (Steigen et al., 2015) *Ca*. Similichlamydia labri sp. (8/133) or more distantly related chlamydial sequences only 82% identical to various environmental *Chlamydiae* (Horn and Wagner, 2001) (7/133). Whereas from the latter, we could only amplify 290 bp sequences, we were able to obtain 1089 bp sequences for the novel *Ca*. Similichlamydia sp. and these we have deposited in ENA-EMBL data bank. Each river investigated had its own distribution pattern. Aabach, Emme, Glatt, and Buenz all showed higher numbers of *Ca*. P. salmonis compared to *Ca*. C. salmonicola, similar to the pattern seen in sites in the Rhone catchment (Venoge, Boiron, Aubonne), whereas in the Kander, nearly all (19/21) were *Ca*. C. salmonicola. Although molecular and histological data measured different parameters (presence of DNA vs. morphologically discernable cysts), the sequencing data generally supported the dominance of *Ca*. P. salmonis and *Ca*. C. salmonicola in the brown trout population. This is especially noticeable in the Aabach, where histologically there were no cysts visible. Possible explanations for this finding include that not all Chlamydia infections seem to be visible as cysts and that different gill arches were examined for histology and sequencing, therefore different patterns could be present on different gill arches.

## Discussion

In fish of the genera *Salmo* and *Salvelinus*, three bacterial species inducing epitheliocystis were identified so far; *Ca.* P. salmonis, *Ca.* C. salmonicola and recently *Ca*. Brachiomonas cysticola (Draghi et al., 2004, 2010; Mitchell et al., 2010, 2013; Schmidt-Posthaus et al., 2012; Toenshoff et al., 2012; Contador et al., 2016; Guevara Soto et al., 2016). Consistent with these results, the species we found most frequently infecting brown trout were *Ca.* P. salmonis and *Ca.* C. salmonicola. Molecular data to support this statement are based on a selection of river sites, where well preserved samples could be achieved. To further confirm the presence of chlamydial species in the histologically visible cysts, additional studies dealing with immunohistochemistry or *in-situ* hybridization would be necessary, although in previous studies we could unequivocally demonstrate the presence of *Ca.* P. salmonis and *Ca.* C. salmonicola in the cysts because of typical morphological features using transmission electron microscopy (Schmidt-Posthaus et al., 2012; Guevara Soto et al., 2016).

In this survey, we could demonstrate a wide distribution of epitheliocystis infections in the upper catchments of two major rivers in Europe, the Rhine and the Rhone, indicating that these agents are endemic in the upper reaches of these two river systems. Infections were found more often and were more intensive in the Rhine tributaries compared to the Rhone catchment. Molecularly, the epitheliocystis agents found in the Rhine and the Rhone catchments were similar. Additionally, *Ca*. P. salmonis in Swiss brown trout were the same as previously identified in salmonids in the North Sea (Draghi et al., 2004). Up to about 100 years ago, Atlantic salmon (*S. salar*) were still migrating from the North Sea and the Atlantic Ocean into the alpine regions of Switzerland for spawning (Mertens et al., 2011). Since then, rivers were remodeled and construction of power stations blocked the way up the Rhine into the Swiss tributaries. Therefore, if salmon are the primary hosts, then the epitheliocystis agents must have been exchanging between the North Sea and the Rhine headwaters via migrating salmon in the past. This would not explain the similar distributions in the headwaters of the Rhone, which is rather indicating an exchange of trout between rivers of the Rhine and Rhone catchments, possibly due to translocation of trout. However, the river-specific proportions of chlamydial species seen in our study indicate a more recent site and/or river specific development of chlamydial infections. This locally specific pattern could be seen before in our recent studies in single river systems in the Rhine as well as in the Rhone catchment (Schmidt-Posthaus et al., 2012; Guevara Soto et al., 2016). Such a distribution would be consistent with local populations of *Salmo trutta fario* as the primary hosts, which can be either resident within stretches of rivers or have restricted migration patterns between river sites and the connected lakes. Additionally, there are no salmon farms in Switzerland so far and no resident wild salmon populations which could serve as a source of infection. To investigate possible environmental factors influencing distribution of chlamydial species, their prevalence or infection intensities, further studies will be needed which will investigate differences between involved rivers in possible stress factors like water quality, habitat structure, or water temperature.

Maximal sampling size per river site was 25 animals, therefore negative results have a limited statistical reliability. However, we could show that the epitheliocystis infection is widely distributed in Swiss rivers with highly variable prevalence, infection intensities and proportions of identified bacterial species. These results are only applicable to the time point of sampling and no data are available so far about the temporal development of the disease during different years. To investigate this phenomenon, consecutive samplings at the same river sites at the same time points would be necessary.

A transmission of the epitheliocystis agents from wild brown trout downstream to farmed salmon or other farmed salmonid species and vice versa is possible and a reservoir status of the brown trout for epitheliocystis species typical for salmonid species can be hypothesized. This hypothesis is supported by the fact that infections in the brown trout are only associated with mild pathological lesions, especially for infections with *Ca*. C. salmonicola. This indicates a long-lasting co-evolution resulting in a well-balanced host-pathogen relation. Infections with *Ca*. P. salmonis and mixed infections were leading to more severe pathology as was also shown before (Schmidt-Posthaus et al., 2012; Guevara Soto et al., 2016), but in most cases it is unlikely that these infections will induce clinical signs and elevated mortality. To investigate if brown trout are the only reservoirs for these chlamydial species in the headwater regions of the Rhine and Rhone or if other salmonid species can also serve as reservoir, additional investigations into other salmonid species naturally inhabiting these rivers would be necessary. Even if infections with *Ca*. P. salmonis and *Ca*. C. salmonicola seem to be specific for salmonids, other fish species or even invertebrates could serve as transport hosts for the bacteria. However, until now, there are no studies investigating this hypothesis.

The fact that we found genetically identical agents in the Rhine and the Rhone catchment can be due to transmission of the bacteria between Rhine and Rhone catchments via translocation of fish or passively via human activities during recent times. Stocking of brown trout in Swiss rivers is practiced since several decades and translocation of animals between different catchments was likely in the past (Bernet et al., pers. comm.). The Rhone is eventually draining into the Mediterranean, where neither *Ca*. P. salmonis nor *Ca*. C. salmonicola nor the salmonid hosts have been identified so far. However, the infection status in the downstream areas of the Rhone itself and its tributaries is not known. These rivers are inhabited by salmonids known as hosts for the identified chlamydial species, like brown trout.

In two rivers, Emme and Buenz, a high diversity of chlamydial species was found with uncultured Neochlamydia and *Ca*. Similichlamydia sp. in the subset of bacteria identified molecularly. This is the first report of *Ca*. Similichlamydia sp. infections in brown trout and this bacterial species was not identified in freshwater so far. Until now, reports of infections with *Ca*. Similichlamydia sp. are restricted to marine hosts and no infections in salmonids have been described (Stride et al., 2013a,b; Steigen et al., 2015). The closest relative to the strain found in our study is *Ca*. Similichlamydia labra sp. nov., a species recently identified in ballan wrasse used as cleaner fish on Atlantic salmon in sea water cages (Steigen et al., 2015). Infections of salmon in the same environment are not mentioned. To further characterize the new chlamydial species found in brown trout in our study, in-depth investigations in the phylogenetic relationship will be necessary. The sequences of the strains found in our study have been deposited with EMBL under the following accession numbers: LT222046–LT222048. In this respect, it is worth noting that PCR alone is not a suitable method to distinguish agents of chlamydial infections in brown trout, as primers are directed against a general Chlamydial signature sequence. However, for screening purposes the use of even the short 290 bp sequence is a practical and efficient method (Everett et al., 1999; Guevara Soto et al., 2016). This also gives the advantage of detecting all Chlamydial agents causing epitheliocystis and not being restricted to single bacterial species. To confirm the results and identify the bacterial species involved, sequencing of the PCR products, either directly or after cloning, is always necessary.

In the past, epitheliocystis infections were correlated to water temperature with a seasonal occurrence mainly during summer and autumn (Schmidt-Posthaus et al., 2012). However, in the present study, no correlation could be found between water temperature and prevalence or infection intensity, although the sampling represents “snapshots,” and we were not able to repeatedly sample the same sites throughout the year. In this study, the latest investigations in the year were performed in November with positive fish found at this time point. Therefore, it is still unclear whether epitheliocystis incidences in brown trout continue throughout the winter months and provide a reservoir for the following year. Temperature independence of epitheliocystis infections, namely infections with *Ca*. B. cysticola, were also seen in lake trout (*S. namaycush*, Walbaum) raised for stocking. Contador et al. (2016) describe infection and mortality peaks during winter months with mortality reaching up to 40%. Further studies investigating the same populations of brown trout throughout the year, along with other fish within the same river sites, are clearly necessary if we are aiming to gain an in-depth understanding into the propagation of this disease.

## Author contributions

MG, data acquisition; interpretation of data for the work; drafting the work and revising it for important intellectual content; final approval of the version to be published; agreement to be accountable for all aspects of the work in ensuring that questions related to the accuracy or integrity of any part of the work are appropriately investigated and resolved; LV, experimental design, hypotheses; molecular biology analyses; interpretation of data for the work, experimental design, and organization, data analyses; revised the work for important intellectual content; final approval of the version to be published, agreement to be accountable for all aspects of the work in ensuring that questions related to the accuracy or integrity of any part of the work are appropriately investigated and resolved; HS, revised the work for important intellectual content; final approval of the version to be published; agreement to be accountable for all aspects of the work in ensuring that questions related to the accuracy or integrity of any part of the work are appropriately investigated and resolved; TW, revised the work for important intellectual content; final approval of the version to be published; agreement to be accountable for all aspects of the work in ensuring that questions related to the accuracy or integrity of any part of the work are appropriately investigated and resolved; BV, statistical analyses, interpretation of data for the work; revised the work for important intellectual content; final approval of the version to be published; agreement to be accountable for all aspects of the work in ensuring that questions related to the accuracy or integrity of any part of the work are appropriately investigated and resolved; HSP, experimental design and organization, hypotheses; interpretation of data for the work; drafting the work; revised the work for important intellectual content; final approval of the version to be published; agreement to be accountable for all aspects of the work in ensuring that questions related to the accuracy or integrity of any part of the work are appropriately investigated and resolved.

## Funding

This study was partly financed by the ESKAS - Bundesstipendien für ausländische Studierende, Kunstschaffende und Forschende, Bern, Switzerland.

### Conflict of interest statement

The authors declare that the research was conducted in the absence of any commercial or financial relationships that could be construed as a potential conflict of interest.
